# Association Between Hip Bone Mineral Density and Mortality Risk After Hip Fracture: A Prospective Cohort Study

**DOI:** 10.1007/s00223-023-01109-9

**Published:** 2023-06-22

**Authors:** Yufeng Ge, Yimin Chen, Gang Liu, Shiwen Zhu, Bo Li, Maoyi Tian, Jing Zhang, Xinbao Wu, Minghui Yang

**Affiliations:** 1 Department of Orthopaedics and Traumatology, Peking University Fourth School of Clinical Medicine, Beijing Jishuitan Hospital, Beijing, China; 2 The George Institute for Global Health at Peking University Health Science Centre, Beijing, China; 3 School of Population Health, University of New South Wales, Sydney, NSW Australia

**Keywords:** Hip fracture, Osteoporosis, Bone mineral density (BMD), Mortality, Quantitative computed tomography (QCT)

## Abstract

Factors related to mortality after osteoporotic hip fracture (HF) have been investigated intensively, except for proximal femoral bone mineral density (BMD), which is also the primary cause of osteoporosis. In this study, we aimed to investigate the association of hip BMD with mortality risk after HF. Four hundred and eleven elderly patients with HF in Beijing, China, were included and prospectively followed up with a median time of 3 years. At baseline, quantitative CT technique (QCT) was used to measure areal BMD (aBMD) of the unaffected hip. Areal BMDs of the total hip (TH), femoral neck (FN), trochanter (TR), and intertrochanter were analyzed with postoperative mortality as the primary outcome. A total of 394 patients (78.59 ± 7.59 years, 75.4% female) were included in our final analysis, with 86 (82.23 ± 7.00 years, 81.4% female) dead. All hip bone densities demonstrated a significant association with mortality risks in the unadjusted model, but only TR aBMD remained significantly correlated after adjusting for all covariates. Compared to the lower TR aBMD group, the higher TR aBMD group yielded significantly lower death risks (HR 0.21 95% CI 0.05–0.9, *P* = 0.036). Higher survival probabilities were observed for higher TH and TR aBMD in survival analysis (*P* < 0.001). Hip BMD, especially TR BMD assessed by QCT, is an independent risk factor for postoperative mortality following HF. QCT may present a promising avenue for opportunistic analysis in immobilized patients, providing valuable information for early detection and personalized interventions to enhance patient outcomes.

**Supplementary Information:**

The online version contains supplementary material available at 10.1007/s00223-023-01109-9.

## Introduction

Osteoporotic hip fracture (HF) is a common and severe injury among older people, most of whom suffer from low-energy trauma, imposing a huge burden on patients and health systems [1]. According to the World Health Organization, the number of osteoporotic HF will triple over the next 50 years, from 1.7 million in 1990 to 6.3 million in 2050 worldwide [2]. The injured elderly are usually frail, and the associated comorbidities are known to be implicated in the postoperative risk of death [3, 4]. The one-year mortality rate for HF patients was reported to be up to 20–24%, and the mortality risk may persist beyond 5 years [5]. Thus, it is crucial to identify those patients at high risk of death, which precedes the subsequent interventions and strategies for improvement.

Osteoporosis, the primary cause of osteoporotic HF, is characterized by low bone density, which might be a non-specific marker for aging and weakness. Multiple studies have found that bone mineral density (BMD) is inversely associated with adverse events, including death [6–9]. Lower BMD was further revealed to contribute to higher refracture and death risk after fragility fractures [10], although the evidence was quite limited. Perhaps due to the difficulty for HF elderly patients to examine BMD, few studies focused on the impact of baseline BMD on future mortality after HF, which is still unclear.

Nowadays, to better evaluate the injury pattern and plan the operation, preoperative computed tomography (CT) scans of the proximal femur have become routine in HF patients. The advent of the quantitative CT (QCT) technique further enables radiologists and orthopedists opportunity to explore the hip structure and bone densities [11]. Different parts of the proximal femur could be separated through QCT, and the BMD of some parts (like trochanter (TR) and intertrochanter (IT)) were suggested to be better parameters in clinical prediction than the femoral neck (FN) or total hip (TH) BMD [12–14], which is most often used as a representative measure of proximal femoral BMD. To our knowledge, no studies have explored the association of BMD levels in different parts of the proximal femur with mortality risk after osteoporotic HF.

In this study, using QCT, we first aimed to investigate the association between hip BMD and mortality risk after HF, and second whether the role of bone density in predicting death varies according to different parts of the proximal femur. We hypothesized that higher BMD was associated with lower death risk, and further, TR BMD was a stronger predictor of the mortality risk for HF patients than FN or TH BMD.

## Materials and Methods

### Design

The present study was performed in a single tertiary hospital in Beijing, China, with a co-managed orthogeriatric hip fracture care path. Ethics approval was obtained from the Institutional Review Board of the hospital (201,807-II) before the initiation of this study. The present post hoc analysis used baseline data prospectively collected in our previous observational study [15], exploring the effect of a co-management care model on older hip fracture patients in China (Clinical Trials.gov Identifier: NCT03184896). For our specific purposes, from November 2018 to November 2019, a total of 565 hip fracture subjects over 65 with eligible QCT imaging were screened. The clinical approach has been described previously [15]. All included subjects were followed up by telephone for a median time of 3.5 years (from 2018–2019 to 2021–2022).

During the screening, we ruled out the subjects presented over 48 h from injury to ensure immediate CT imaging after a fracture. We excluded those who cannot fully ambulate and sustained non-ground level falls. Those who underwent conservative therapy, those with contralateral hip lesions (like fractures or bone necrosis) that may disturb imaging measurements, and those with severe diseases that may interfere with bone metabolism (like parathyroid diseases) were also excluded. Further exclusion criteria were pathological fractures or terminal malignancies. All patients gave written informed consent.

### QCT Scans and Bone Mineral Density Measurement

To avoid the bias caused by CT machine, two same Toshiba Aquilion 64-slices CT scanners (Toshiba Medical Systems Corp., Tokyo, Japan) with a solid phantom (Mindways Software Inc., Austin, TX, USA) were used. The subjects were scanned in the supine position, with the phantom beneath the hip. Hips were scanned from the top of the acetabulum to a level of 3 cm inferior to the lesser trochanter or longer to cover the fractured bone. The scan parameters were the same: 120 Kvp, 125 mAs, 1 mm thickness, 50 cm field of view, and 512 × 512 matrix in spiral reconstruction and standard reconstruction. Subsequently, the acquired CT images were transferred to a QCT workstation and analyzed using the computed tomography X-ray absorptiometry function (CTXA, version 4.2.3) of Mindways QCT pro software (Mindways Software Inc., Austin, TX, USA). The measurement procedure was previously described, and the areal BMD (aBMD, g/cm^2^) was obtained from CTquals DXA [16]. Briefly, two-dimensional projections were generated from the three-dimensional CT dataset after image segmentation and proper manipulation. All subjects’ aBMD of the femoral neck (FN), greater trochanter (TR), intertrochanter (IT), and total hip (TH) were then measured on the unaffected side.

### Data Collection and Outcome Measures

The demographic information and perioperative records were prospectively collected. The demographic data included age, sex, weight, height, bone mass index (BMI), drinking or smoking habits, education level, and living status. Baseline medical situations, such as hypertension, diabetes, and cognitive and visual impairment, were also investigated and documented. Mini-Mental State Examination—China (MMSE) [17] was further used to quantify cognitive ability, and participants with an MMSE score of 23 or lower were defined as having cognitive impairment. The Charlson comorbidity index (CCI) [18] was then calculated to further represent the overall medical situation. Perioperative variables included fracture pattern, side, American Society of Anesthesiologists (ASA) scores, type of anesthesia and operation, length of stay (LOS), and rehabilitation or not. Albumin level was also recorded to indicate nutrition status while falling times in the past year indicate the tendency to be injured. The educational level was composed of five states, ranging from illiterate to university or higher. Concerning operations, intertrochanteric fractures were treated by intramedullary nailing, while femoral neck fractures were treated by cannulated screw fixation or arthroplasty. The operation type was categorized into internal fixation (intramedullary nailing and screw fixation) and arthroplasty.

All patients received telephone visits by orthopaedists after 3 years. Our primary outcome was the postoperative three-year mortality from all causes. Secondary outcomes included the living place alteration (institutionalized or not) and health-related quality of life (HRQoL) at the final follow-up. Institutionalized sites refer to hospitals, rehabilitation centers, or nursing homes. Euro-Qol 5 Dimensions Score (EQ-5D 5L) [18], a generic health-utility instrument, was used to measure HRQoL. It consists of a five-level response (no problems, slight problems, moderate problems, severe problems, extreme problems) for five health domains related to daily activities: mobility; self-care; usual activities; pain and discomfort; and anxiety and depression. We then converted the responses into an overall score using a published utility model for the China population [19].

### Statistical Analysis

Data are described as means and standard deviations for parametric data or as medians and interquartile ranges when the data are not normally distributed. Categorical data are presented using frequencies and numerical distributions. The Chi-squared test was used to assess the differences between the two groups for categorical variables and Student’s *t* test or the Mann–Whitney *U* test for continuous variables, as appropriate (parametric *vs* non-parametric data, respectively).

In our present study, considering the inherent differences in BMD between males and females, we first employed sex-specific *Z*-score normalization to facilitate further analysis and interpretation. The baseline variables that were considered clinically relevant or showed a univariate relationship with outcome were considered covariates. Based on that, multivariate Cox proportional-hazards models were used to estimate the association between the baseline BMDs and risk of death, without and with adjustments for age, sex, BMI, CCI, albumin, MMSE, housebound, fracture type, anesthesia type, and time from injury to operation. To ensure parsimony of the final model, we have transformed the latter seven covariates into a propensity score for death, calculated from a multivariate logistic regression model, given the number of death events available. Kaplan–Meier survival plots were produced using TH aBMD and FN aBMD stratified into standardized score categories: Z < − 1; Z ≥ − 1 and Z < 1; and Z ≥ 1. Subgroup analysis were also performed according to age group (above and below 80 years), sex, BMI (above and below 24), and fracture type.

All the analyses were performed with the statistical software packages R 4.1.1 (http://www.R-project.org, The R Foundation). A two-tailed test was performed, and *P* < 0.05 was considered statistically significant.

## Results

### Population and Baseline Characteristics

**Fig. 1:**
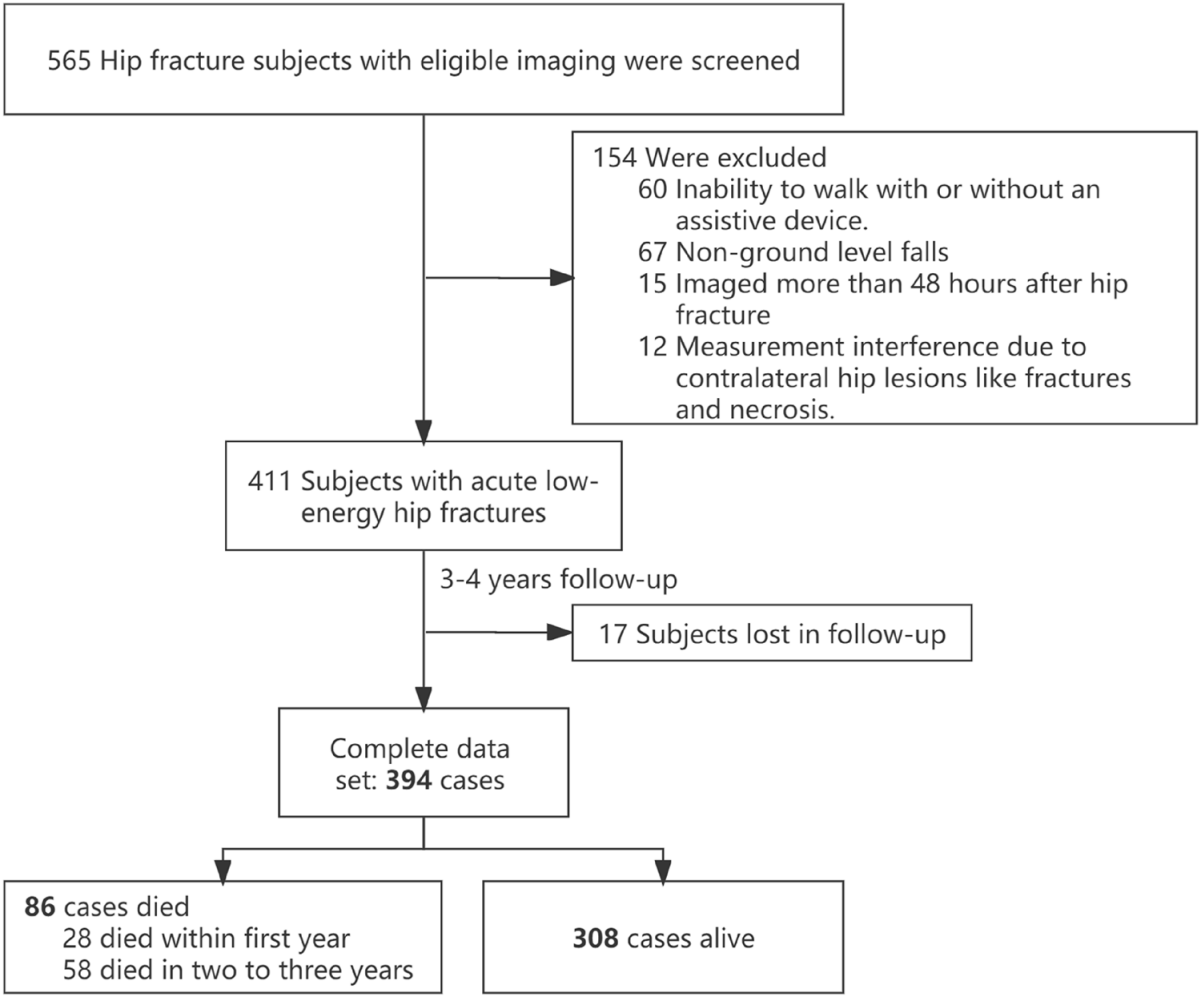
Flowchart of the study

A total of 394 patients were ultimately included in our final analysis after 154 were excluded and 17 dropped out. At the end of the follow-up, 86 (21.8%) of these patients had died, including 28 (7.11%) deaths that occurred in the first year. Figure 1 presents a flowchart of the study.

The baseline characteristics of all available subjects are listed in Table 1. The mean age was 78.59 ± 7.59 years; 297 (75.4%) were women, and 189 (48%) were ITF. The median time from injury to operation was around 3 days, and the median length of stay (LOS) was around 5 days. As shown in Table 1, compared with the groups who died, all bone densities were statistically significantly higher in surviving patients (*P* < 0.001). More patients with cognitive impairment and lower level of albumin were also revealed in the died (26 (30.6%) vs 57 (18.6%); 39.40 ± 3.68 vs 41.36 ± 2.86, respectively).

**Table 1:** Baseline characteristics

Characteristic	Total (*n* = 394)	Alive (*n* = 308)	Died (*n* = 86)	*P* value
Age, years, Mean ± SD	78.59 ± 7.59	77.58 ± 7.44	82.23 ± 7.00	< 0.001
Female, *n* (%)	297 (75.4)	227 (73.7)	70 (81.4)	0.143
Weight, kg, Mean ± SD	60.12 ± 11.42	61.22 ± 11.37	56.17 ± 10.76	< 0.001
Height, cm, Mean ± SD	161.42 ± 8.06	161.94 ± 7.98	159.55 ± 8.09	0.015
BMI, kg/m^2^, Mean ± SD	23.00 ± 3.63	23.27 ± 3.55	22.03 ± 3.75	0.005
CCI, *n* (%)				0.692
0	113 (28.7)	90 (29.2)	23 (26.7)	
1	149 (37.8)	119 (38.6)	30 (34.9)	
2	77 (19.5)	59 (19.2)	18 (20.9)	
≥ 3	55 (14.0)	40 (13)	15 (17.4)	
Diabetes, *n* (%)	130 (33.0)	111 (36)	19 (22.1)	0.015
Hypertension, *n* (%)	257 (65.2)	203 (65.9)	54 (62.8)	0.591
Ever or current smokers, *n* (%)	60 (15.96)	47 (15.67)	13 (17.11)	0.896
Current drinkers, *n* (%)	28 ( 7.1)	23 (7.5)	5 (5.8)	0.598
Educational level, *n* (%)				0.199
Illiterate	77 (19.5)	55 (17.9)	22 (25.6)	
Primary school or lower	95 (24.1)	71 (23.1)	24 (27.9)	
High school	163 (41.4)	133 (43.2)	30 (34.9)	
University or higher	59 (15.0)	49 (15.9)	10 (11.6)	
MMSE scale, Mean ± SD	25.60 ± 4.94	25.93 ± 4.65	24.39 ± 5.73	0.010
Cognitive impairment, *n* (%)	83 (21.2)	57 (18.6)	26 (30.6)	0.017
Live alone, *n* (%)	54 (13.7)	38 (12.3)	16 (18.6)	0.135
Live at home, *n* (%)	390 (99.0)	304 (98.7)	86 (100)	0.581
Housebound, *n* (%)	73 (18.5)	51 (16.6)	22 (25.6)	0.057
Visual impairment, *n* (%)	158 (40.1)	130 (42.2)	28 (32.6)	0.106
Albumin, g/L, Mean ± SD	40.93 ± 3.16	41.36 ± 2.86	39.40 ± 3.68	< 0.001
Falling times in the past year, *n* (%)				0.047
0	162 (41.1)	131 (42.5)	31 (36)	
1	177 (44.9)	141 (45.8)	36 (41.9)	
≥ 2	55 (14.0)	36 (11.7)	19 (22.1)	
Time from injury to operation, days, Median (IQR)	3.0 (2.0, 4.0)	3.0 (2.0, 4.0)	3.0 (2.0, 4.0)	0.352
Fracture type, *n* (%)				0.247
FNF	205 (52.0)	165 (53.6)	40 (46.5)	
ITF	189 (48.0)	143 (46.4)	46 (53.5)	
Fracture side, *n* (%)				0.165
Left	191 (48.5)	155 (50.3)	36 (41.9)	
Right	203 (51.5)	153 (49.7)	50 (58.1)	
Anesthesia type, *n* (%)				0.759
Spinal anesthesia	378 (95.9)	296 (96.1)	82 (95.3)	
General	16 ( 4.1)	12 (3.9)	4 (4.7)	
ASA, *n* (%)				0.142
I	56 (14.2)	47 (15.3)	9 (10.5)	
II	219 (55.6)	175 (56.8)	44 (51.2)	
III	119 (30.2)	86 (27.9)	33 (38.4)	
Operation type, *n* (%)				0.348
Internal fixation	230 (58.4)	176 (57.1)	54 (62.8)	
Arthroplasty	164 (41.6)	132 (42.9)	32 (37.2)	
Physiotherapy, *n* (%)	281 (71.3)	216 (70.1)	65 (75.6)	0.323
LOS, days, Median (IQR)	5.0 (4.0, 6.0)	5.0 (4.0, 6.0)	5.0 (4.0, 6.0)	0.522
Bone mineral density				
TH aBMD, g/cm^2^, Mean ± SD	0.57 ± 0.12	0.59 ± 0.12	0.52 ± 0.10	< 0.001
TR aBMD, g/cm^2^, Mean ± SD	0.40 ± 0.10	0.41 ± 0.10	0.35 ± 0.08	< 0.001
FN aBMD, g/cm^2^, Mean ± SD	0.50 ± 0.11	0.51 ± 0.11	0.46 ± 0.09	< 0.001
IT aBMD, g/cm^2^, Mean ± SD	0.70 ± 0.15	0.71 ± 0.15	0.64 ± 0.12	< 0.001

*BMI* bone mass index, *CCI* Charlson’s comorbidity index, *MMSE* mini-mental state examination, *FNF* femoral neck fracture, *ITF* intertrochanteric fracture, *ASA* American society of anesthesiologists, *LOS* length of stay, *TH aBMD* total hip areal bone mineral density, *TR aBMD* trochanter areal bone mineral density, *FN aBMD* femoral neck areal bone mineral density, *IT aBMD* intertrochanter areal bone mineral density

### Primary Outcome

**Fig. 2:**
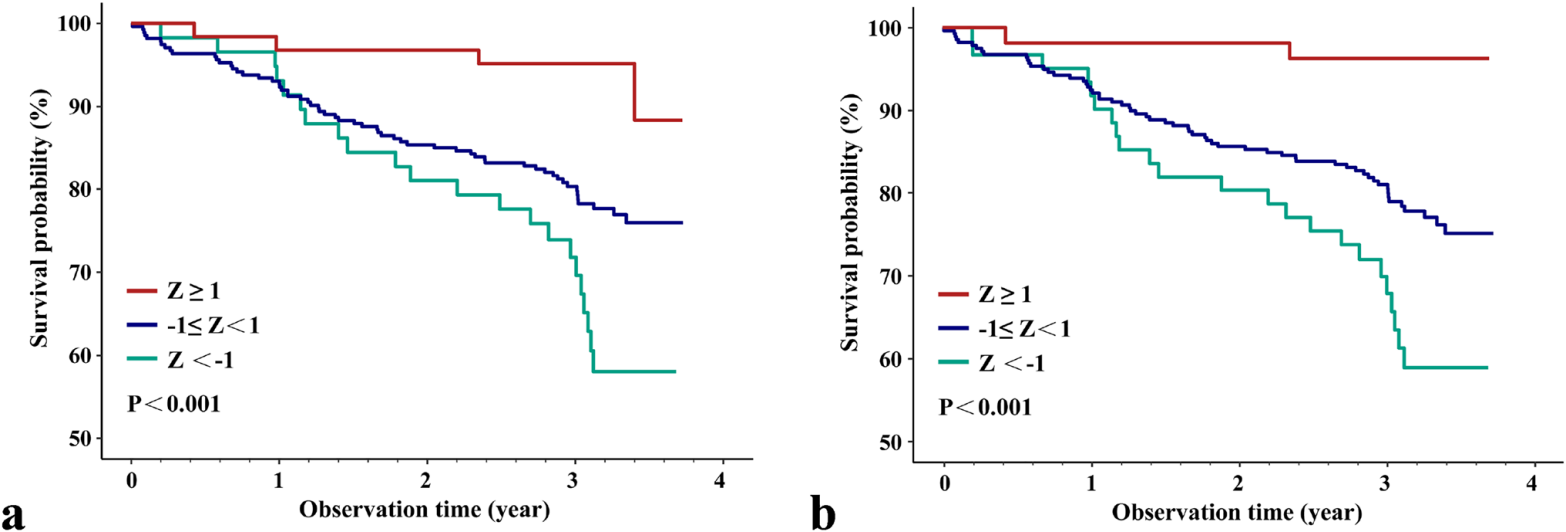
Kaplan–Meier curves for probability of death stratified by sex-specific THaBMD and TRaBMD. **a** TH aBMD, **b** TR aBMD, *TH aBMD* total hip areal bone mineral density, *TR aBMD* trochanter areal bone mineral density

In the unadjusted model, all hip bone densities were found to be significantly associated with the risk of death following hip fractures, as shown in Table 2. However, after adjusting for age, sex, and BMI in Model 1, only TH aBMD (HR 0.75, 95% CI 0.59–0.96, *P* = 0.024) and TR aBMD (HR 0.7, 95% CI 0.54–0.9, *P* = 0.006) remained significantly correlated with mortality risk. Further adjustment for all covariates in Model 2 revealed that TR aBMD was still significantly linked to postoperative mortality. Compared to the lower TR aBMD group (Z < − 1), the higher TR aBMD group (Z ≥ 1) yielded significantly lower death risks (HR 0.21 95% CI 0.05–0.9, *P* = 0.036). Kaplan–Meier curve also demonstrated lower survival probability in lower TH and TR aBMD groups (Fig. 2). Subgroup analysis examined the interaction and verified the robust associations (Supplementary Fig. 3).

**Table 2:** HRs of bone parameters for 3-year mortality

	Died vs alive (86 vs 308)					
Bone parameters	Unadjusted		Model 1		Model 2	
	HR (95% CI)	*P* value	HR (95% CI)	*P* value	HR (95% CI)	*P* value
TH aBMD* (g/cm^2^)	0.61 (0.48 ~ 0.77)	< 0.001	0.75 (0.59 ~ 0.96)	0.024	0.83 (0.65 ~ 1.06)	0.127
TR aBMD* (g/cm^2^)	0.57 (0.44 ~ 0.73)	< 0.001	0.7 (0.54 ~ 0.9)	0.006	0.79 (0.61 ~ 1.03)	0.085
FN aBMD* (g/cm^2^)	0.67 (0.52 ~ 0.85)	0.001	0.81 (0.64 ~ 1.03)	0.084	0.85 (0.67 ~ 1.07)	0.16
IT aBMD* (g/cm^2^)	0.66 (0.53 ~ 0.83)	< 0.001	0.82 (0.65 ~ 1.04)	0.096	0.88 (0.7 ~ 1.11)	0.291
TH aBMD Category						
Z < − 1	Reference		Reference		Reference	
− 1 ≤ Z < 1	0.56 (0.34 ~ 0.92)	0.021	0.83 (0.49 ~ 1.41)	0.496	0.92 (0.55 ~ 1.55)	0.754
Z ≥ 1	0.15 (0.05 ~ 0.44)	0.001	0.31 (0.1 ~ 0.95)	0.04	0.35 (0.12 ~ 1.07)	0.067
TR aBMD Category						
Z < − 1	Reference		Reference		Reference	
− 1 ≤ Z < 1	0.55 (0.34 ~ 0.89)	0.015	0.81 (0.48 ~ 1.34)	0.409	0.97 (0.58 ~ 1.63)	0.914
Z ≥ 1	0.09 (0.02 ~ 0.36)	0.001	0.15 (0.04 ~ 0.66)	0.012	0.21 (0.05 ~ 0.9)	0.036
FN aBMD Category						
Z < − 1	Reference		Reference		Reference	
− 1 ≤ Z < 1	0.51 (0.3 ~ 0.85)	0.01	0.81 (0.47 ~ 1.41)	0.455	0.93 (0.53 ~ 1.62)	0.796
Z ≥ 1	0.28 (0.12 ~ 0.66)	0.004	0.52 (0.21 ~ 1.29)	0.159	0.57 (0.23 ~ 1.43)	0.233
IT aBMD Category						
Z < − 1	Reference		Reference		Reference	
− 1 ≤ Z < 1	0.51 (0.31 ~ 0.84)	0.008	0.74 (0.44 ~ 1.26)	0.267	0.88 (0.52 ~ 1.49)	0.636
Z ≥ 1	0.32 (0.14 ~ 0.72)	0.006	0.62 (0.26 ~ 1.47)	0.28	0.73 (0.31 ~ 1.73)	0.472

Model 1, adjusted for age, sex, and bone mass index;

Model 2, adjusted for Model 1 + propensity score (calculated by CCI, albumin, MMSE, housebound, fracture type, anesthesia type, and time from injury to operation);

*TH aBMD* total hip areal bone mineral density, *TR aBMD* trochanter areal bone mineral density, *FN aBMD* femoral neck areal bone mineral density, *IT aBMD* intertrochanter areal bone mineral density;

*Per sex-specific SD increase

### Secondary Outcome

In terms of the HRQoL and living place alteration, higher BMD was associated with higher EQ-5D and a lower risk of institutionalization (Table 3). However, the significant association faded away to varying degrees after adjustments.

**Table 3:** Effect size of bone parameters for secondary outcome

	EQ-5D				Institutionalized vs home (19 vs 321)			
Bone parameters	Unadjusted		Model 1		Unadjusted		Model 1	
	B (95% CI)	*P* value	B (95% CI)	*P* value	OR (95% CI)	*P* value	OR (95% CI)	*P* value
TH aBMD* (g/cm^2^)	0.0526 (0.012–0.0933)	0.012	0.0256 (− 0.0166–0.0677)	0.235	0.59 (0.36–0.99)	0.044	0.75 (0.42–1.32)	0.321
TR aBMD* (g/cm^2^)	0.0619 (0.022–0.1017)	0.003	0.033 (− 0.0088–0.0748)	0.123	0.54 (0.31–0.93)	0.027	0.65 (0.35–1.22)	0.18
FN aBMD* (g/cm^2^)	0.0315 (− 0.0091–0.0722)	0.13	0.0102 (− 0.0308–0.0512)	0.626	0.47 (0.26–0.84)	0.011	0.55 (0.3–1.03)	0.064
IT aBMD* (g/cm^2^)	0.0475 (0.0067–0.0883)	0.023	0.022 (− 0.0201–0.0641)	0.307	0.75 (0.46–1.21)	0.238	0.97 (0.57–1.66)	0.92

EQ-5D was followed up in 308 alive participants, and data of living place alteration were missed in 50 participants after excluding four participants who lived in nursing home before injury

Model 1, adjusted for age, sex, and bone mass index;

*TH aBMD* total hip areal bone mineral density, *TR aBMD* trochanter areal bone mineral density, *FN aBMD* femoral neck areal bone mineral density, *IT aBMD* intertrochanter areal bone mineral density; *EQ-5D* Euro-Qol 5 dimensions;

*Per sex-specific SD increase

## Discussion

In the proximal femur, trochanter (TR) BMD performs better in representing femoral strength and predicting second hip fracture than FN or TH BMD [12, 14]. However, few studies have focused on its potential in forecasting risks of death after a first HF. As far as we know, this is the first study to explore the different roles of proximal femoral BMD in predicting the mortality risks after HF occurrence in the elderly. The study results demonstrated hip BMD as a risk factor for postoperative mortality and revealed that TR aBMD was the most relevant parameter in predicting mortality risks. Higher TR aBMD and TH aBMD yielded much lower risks of death during three-year follow-up after a first HF in the elderly.

Our study found an inverse association between hip bone densities and postoperative mortality probabilities, which aligns with many previous researches [10, 20, 21]. Little explanations were given, and the intrinsic connections were still unclear. A recent systematic review [22] stated that bisphosphonates reduce all-cause mortality after osteoporotic fractures, which indirectly reflected the effect of bone density on postoperative mortality. Generally, skeletal health is a pretty good marker for overall health status, and low BMD is related to multiple factors, like estrogen metabolism [23], nutrition [24], as well as genetic factors [25]. Indeed, a range of risk factors was shared in the pathophysiological mechanisms of bad habits, chronic diseases, and low BMD, such as aging, smoking, and lack of physical activity. The albumin level relating to the overall nutrition status and the MMSE score relating to the cognitive ability were also lower in the dead group in our study, which is consistent with other studies [26, 27]. The potential metabolic association between the comorbidities, adverse events, and BMD might suggest that the bone parameters could give a better hint or predict the prognosis after surgery, as in our present study.

Moreover, we found that the TR BMD plays a prominent role in hip BMD predicting mortality. The greater trochanter is the primary attachment of posterior hip muscles (gluteus), which are strong muscles that help maintain gait stability and standing balance. The constant mechanical loading and physical forces created by the gluteus contractions could partly explain that TR BMD might better represent the proximal femur strength, as Cheng demonstrated in his in-vitro study [12]. Yin et al. [28] found a significant association between trochanter BMD and gluteus maximus muscle area through QCT imaging. In addition, TR aBMD was discovered to be the only bone parameter tightly associated with handgrip strength [28], which has become a critical indicator for screening sarcopenia [29]. The development of osteosarcopenia has suggested an indissoluble underlying crosstalk between bone and muscle metabolism [30]. It is reasonable to hypothesize that TR aBMD could be an optimal parameter predicting overall health and physical performance, which needs to be verified in well-powered studies. Furthermore, lower TR aBMD was linked to a higher probability of intertrochanteric fracture (ITF) [13], which was also a risk factor for death [4]. However, following adjustment for key confounding factors, including fracture type, CCI, nutrition level, cognitive status, and anesthesia type [4, 31], TR aBMD remained a strong and independent risk factor for postoperative death.

Few studies have explored the relationship between bone density and mortality in the context of postoperative death after HF. As described in some studies [32–35], this may be due to the challenges of obtaining dual-energy X-ray absorptiometry (DXA) scans in the acute setting for HF patients. Radiological parameters (such as cortical thickness [34, 35], Singh index, and Dorr classification [32]) were revealed to be associated with osteoporosis or mortality risks. Despite this, lots of BMD information from clinical CT imaging of HF has not been fully utilized. The findings in our study demonstrated that TR BMD assessed by QCT may serve as a potential indicator for death after HF using opportunistic analysis of routine clinical CT scans. QCT has its advantages in providing body information for patients with difficulty moving after injury, such as those with HF. We believe that the extensive information provided by routine CT scans can add tremendous value to patient care in the future [11, 36].

One of the potential strengths of our study was its comprehensive, prospectively collected data, ensuring minimal recall bias. During inclusion, we only selected those who could fully ambulate and excluded those who presented more than 48 h after injury to minimize the influence of immobilization on BMD. Besides, the well-documented medical information and adjusted confounding bias further helped us to draw a more robust relationship between bone densities and mortality. This study also has several limitations. First, the mortality rate over 3 years in our study was found to be 21.8%, which is notably lower than that reported in the previous studies [5, 37]. This could be attributed to the implementation of our co-management care model, which may effectively mitigate the mortality risks [15, 38]. Also, our study participants were relatively younger and exhibited better mobility [5]. Therefore, caution should be taken when comparing our findings with others. Second, the sample size of the cohort was relatively small, which also imposed restrictions on our analysis. Even so, significant associations between BMD and mortality were detected. Further well-designed studies with a larger sample size may dig deeper into the role of bone parameters in predicting HF patients’ prognosis.

In conclusion, hip BMD, especially TR BMD assessed by QCT, is an independent risk factor for postoperative mortality following HF. QCT may present a promising avenue for opportunistic analysis in immobilized patients, providing valuable information for early detection and personalized interventions to enhance patient outcomes.

## Supplementary Information

Below is the link to the electronic supplementary material.

Supplementary file1 (DOCX 171 KB)
