# Supplementary material for: Association Between Hip Bone Mineral Density and Mortality Risk After Hip Fracture: A Prospective Cohort Study
Source: Calcif Tissue Int. 2023 Jun 22;113(3):295–303. doi: 10.1007/s00223-023-01109-9 (PMC10449952; doi:10.1007/s00223-023-01109-9)
Supplement: Supplementary file 1 — Supplementary file1 (DOCX 171 KB) [file 223_2023_1109_MOESM1_ESM.docx]

**
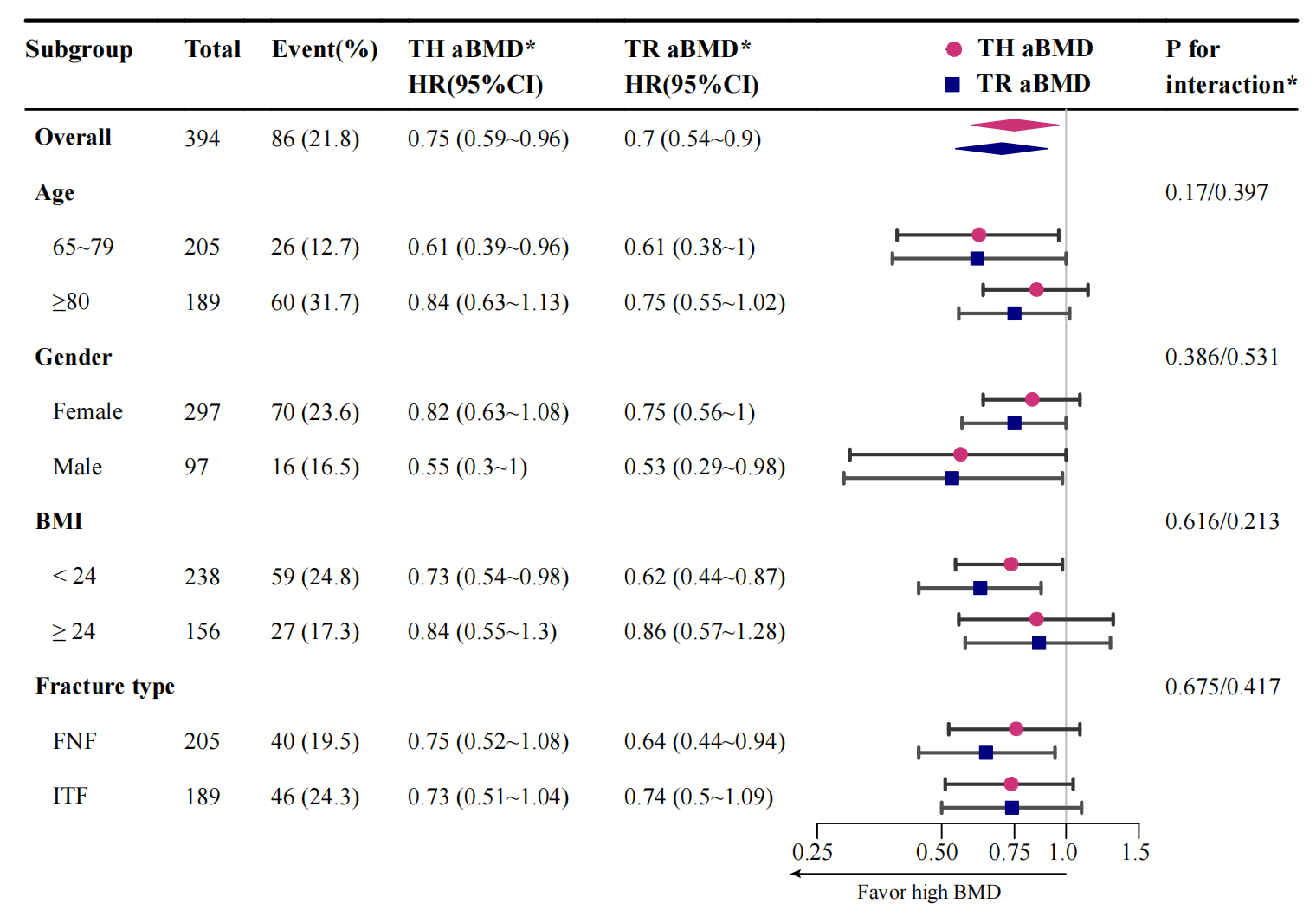
**

**Supplementary Fig.3** HRs of mortality risk per sex-specific SD increase of TH aBMD and TR aBMD in different subgroups.

*the former for TH aBMD and the latter for TR aBMD

*TH aBMD* total hip areal bone mineral density, *TR aBMD* trochanter areal bone mineral density, *BMI* bone mass index, *FNF* femoral neck fracture, *ITF* intertrochanteric fracture;
